# Profiling Anticancer and Antioxidant Activities of Phenolic Compounds Present in Black Walnuts (*Juglans nigra*) Using a High-Throughput Screening Approach

**DOI:** 10.3390/molecules25194516

**Published:** 2020-10-02

**Authors:** Khanh-Van Ho, Anuradha Roy, Sarah Foote, Phuc H. Vo, Namrita Lall, Chung-Ho Lin

**Affiliations:** 1 Center for Agroforestry, School of Natural Resources, University of Missouri, Columbia, MO 65211, USA; vkh6c6@mail.missouri.edu (K.-V.H.); phucvh2410@gmail.com (P.H.V.); namrita.lall@up.ac.za (N.L.); 2 Department of Food Technology, Can Tho University, Can Tho 90000, Vietnam; 3 High Throughput Screening Laboratory, University of Kansas, Lawrence, KS 66047, USA; anuroy@ku.edu; 4 CEVA Biomune, Lenexa, KS 66215, USA; sarah.foote@ceva.com; 5 Department of Plants and Soil Sciences, Plant Science Complex, University of Pretoria, Pretoria 0002, South Africa

**Keywords:** penta-*O*-galloyl-*β*-d-glucose, polyphenol, antioxidant response element

## Abstract

Our recent studies have demonstrated multiple health-promoting benefits from black walnut kernels. These biological functions of black walnuts are likely associated with their bioactive constituents. Characterization of phenolic compounds found in black walnut could point out underexplored bioactive activities of black walnut extracts and promote the development of novel applications of black walnut and its by-products. In the present study, we assessed bioactivity profiles of phenolic compounds identified in the kernels of black walnuts using a high-throughput screening (HTS) approach. Black walnut phenolic compounds were evaluated in terms of their total antioxidant capacity, antioxidant response element (ARE) induction, and anticancer activities. The anticancer activities were identified by evaluating the effects of the phenolic compounds on the growth of the tumorigenic alveolar epithelial cells (A549) and non-tumorigenic lung fibroblast cells (MRC-5). Out of 16 phenolic compounds tested, several compounds (penta-*O*-galloyl-*β*-d-glucose, epicatechin gallate, quercetin, (–)-epicatechin, rutin, quercetin 3-*β*-d-glucoside, gallic acid, (+)-catechin, ferulic acid, syringic acid) exerted antioxidant activities that were significantly higher compared to Trolox, which was used as a control. Two phenolic compounds, penta-*O*-galloyl-*β*-d-glucose and quercetin 3-*β*-d-glucoside, exhibited antiproliferative activities against both the tumorigenic alveolar epithelial cells (A549) and non-tumorigenic lung fibroblast cells (MRC-5). The antioxidant activity of black walnut is likely driven not only by penta-*O*-galloyl-*β*-d-glucose but also by a combination of multiple phenolic compounds. Our findings suggested that black walnut extracts possibly possess anticancer activities and supported that penta-*O*-galloyl-*β*-d-glucose could be a potential bioactive agent for the cosmetic and pharmaceutical industries.

## 1. Introduction

Black walnut (*Juglans nigra* L.) is an economically valuable tree for edible nut production in the United States [1]. This native tree nut constitutes a major part of the nut production industry in the U.S. Midwest, with over 15 million pounds processed annually in Missouri [2]. People traditionally valued black walnut kernels as a health-promoting food source based on its compositional profile and utilized other parts of the trees (e.g., leaves, barks) for multiple medical purposes to treat diarrhea and bilious and cramp colic [3]. Consumption of black walnut kernels has been linked to potential health-promoting activities, such as lowering cholesterol absorption, anti-inflammatory effects, and prevention of certain cancers [4].

Our recent studies have demonstrated a wide range of biological functions of kernel extracts derived from black walnuts including antibacterial, antioxidant, and anti-inflammatory potential [5,6,7]. Ho et al. [6] reported antibacterial capacities of 22 black walnut cultivars selected for nut production by the University of Missouri Center for Agroforestry (Columbia, Missouri, USA) [8]. Several black walnut cultivars (e.g., Mystry, Surprise) exhibited antibacterial activity against a Gram-positive bacterium (*Staphylococcus aureus*) and the antibacterial activities were variable among the tested cultivars. Glansreginin A, azelaic acid, and quercetin were predominant phenolic compounds responsible for the antibacterial activities. These compounds were successfully identified in the bioactive fraction of kernel extracts from Mystry via a bioassay guided purification strategy combined with a metabolomics approach [6].

Black walnut kernels have also been reported to possess anti-inflammatory potential. The kernel extracts of black walnuts exhibited inhibitory effects on the production of several anti-inflammatory mediators (e.g., interleukin (IL)-1β, tumor necrosis factor alpha (TNF-α), monocyte chemoattractant protein (MCP)-1, IL-6, IL-8) in human promonocytic cell line U-937 model system [5]. The cytokine suppressive activities were variable among the black walnut cultivars examined. Two cultivars, Surprise and Sparrow, significantly inhibited the cytokine production of all examined cytokines in the U-937 cells. Additionally, our recent findings revealed antioxidant activities of the kernel extracts derived from six black walnut cultivars. Mystry showed the strongest antioxidant capacities compared with other tested cultivars [7].

Health-promoting properties of black walnuts are likely associated with a wealth of phytochemicals presented in black walnut kernels. Several polyphenols detected in the kernel extracts of black walnut are known to possess a variety of bioactive functions such as anti-inflammatory, antioxidant, antibacterial, and anticancer activities. Our previous studies have identified 17 phenolic compounds in the kernels of 11 black walnut cultivars [6,9] and many of these compounds (e.g., ellagic acid, epicatechin gallate, naringin, penta-*O*-galloyl-*β*-d-glucose, quercetin-3-*β*-d-glucoside) are known to possess antioxidant and anticancer activity [10,11,12].

High-throughput screening (HTS) is a critical tool to expand biomedical knowledge of small molecules that can be used for the drug discovery industry [13]. The high-throughput analytical technologies enable us to evaluate the biological functions of large amounts of chemicals or natural materials in the shortest amount of time by integrating chemical analyses, modeling, and machine learning that can result in marketed pharmaceutical products in the lowest cost production [14]. In this study, we utilized high-throughput screening assays to identify antioxidant and anticancer potentials of phenolic compounds found in black walnuts. The exploration of biological functions of bioactive compounds in black walnuts could reveal underexplored bioactive activities of black walnut extracts and promote the development of novel applications of black walnut and its by-products.

## 2. Results

### 2.1. Total Antioxidant Capacity

**Figure 1:**
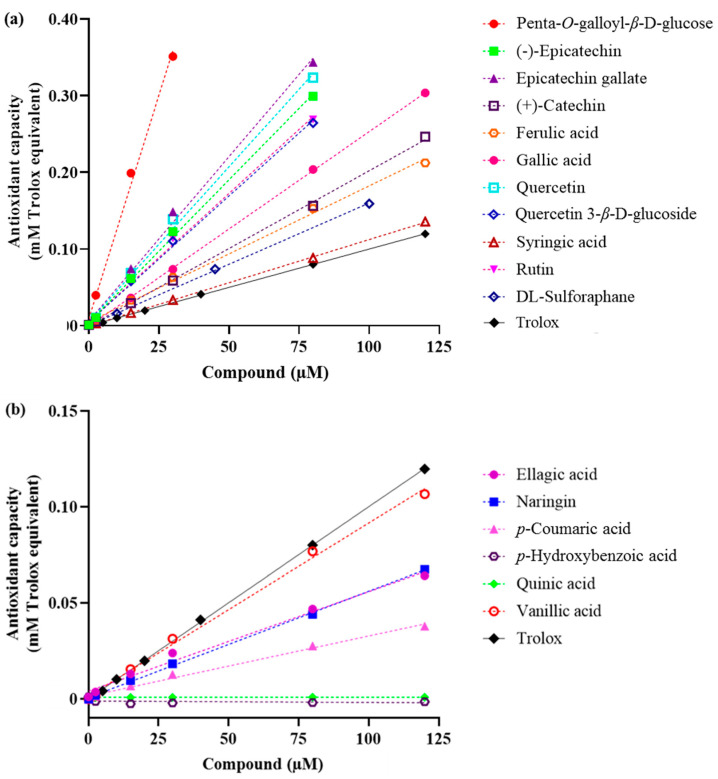
Total antioxidant activity of phenolic compounds in black walnut. (**a**) Compounds with higher antioxidant capacity than Trolox, (**b**) compounds with lower antioxidant capacity than Trolox.

Our previous studies have documented the presence of 17 phenolic compounds in black walnuts [6,9]. Out of 17 phenolic compounds identified, 16 compounds were evaluated for their antioxidant and anticancer activities, whereas the bioactive activities of glansreginin A were not examined since this compound was not commercially available. Out of 16 phenolic compounds tested, 10 compounds (penta-*O*-galloyl-*β*-d-glucose, epicatechin gallate, quercetin, (–)-epicatechin, rutin, quercetin 3-*β*-d-glucoside, gallic acid, (+)-catechin, ferulic acid, syringic acid) exhibited higher total antioxidant capacity compared with the control (Trolox). Six phenolic compounds including vanillic acid, ellagic acid, naringin, *p*-coumaric acid, *p*-hydroxybenzoic acid, and quinic acid had lower total antioxidant capacity than Trolox (Figure 1). The fold-increase over Trolox of these phenolic compounds ranged from 1.1 to 11.5. Penta-*O*-galloyl-*β*-d-glucose exhibited the highest total antioxidant capacity, followed by epicatechin gallate, quercetin, (–)-epicatechin, rutin, quercetin 3-*β*-d-glucoside, gallic acid, (+)-catechin, ferulic acid, and syringic acid, respectively (Table 1).

Linear regression models of the majority of tested compounds had high R^2^ values (>0.98), indicating that these models were reliable (Table 1). Models of two compounds (*p*-hydroxybenzoic acid and quinic acid) had low values of R^2^ since these compounds had minor or no antioxidant capacity under the experimental conditions. Violin plots representing the data distribution of controls (Trolox and tert-butylhydroquinone) showed a relatively small variation of data, indicating that the HTS assay system was reliable (Supplementary Figures S1 and S2).

### 2.2. Antioxidant Response Element (ARE) Activation

**Figure 2:**
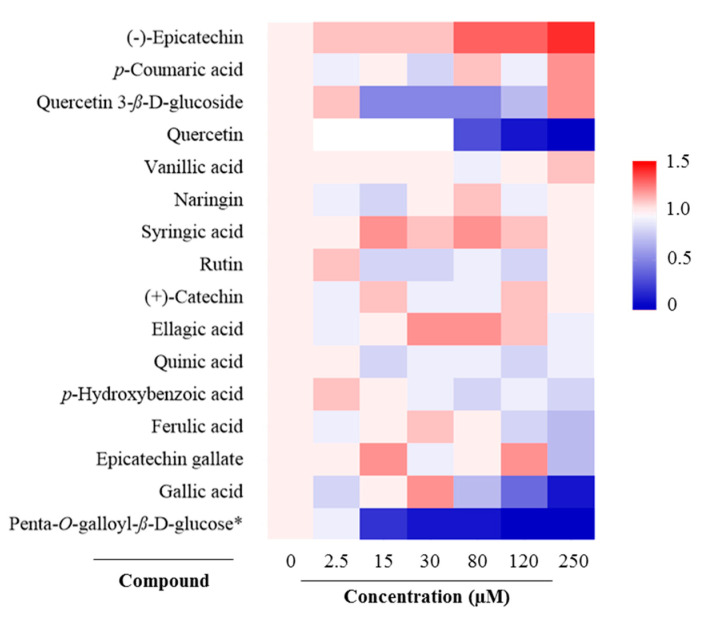
Antioxidant response element (ARE) activation activities of the tested compounds in HepG2-ARE cells. In the heatmap, color represents relative fold-increases in ARE activities in HepG2-ARE cells treated with DMSO and compounds compared with the corresponding vehicle control, the HepG2-ARE cells treated with 0.35% DMSO only. * Penta-*O*-galloyl-*β*-d-glucose was screened at concentrations of 0, 2.5, 15, 30, 80, 125, 175 µM, respectively.

The ARE fold-increase in HepG2-ARE activation of all compounds relative to the control was <2 (Figure 2). Since the ARE fold-increase in HepG2-ARE of examined compounds was <10 [15], there were no compounds that could be considered to exert significant ARE induction activity. (−)-Epicatechin showed the strongest ARE activation among all compounds, followed by *p*-coumaric acid, quercetin 3-*β*-d-glucose, and vanillic acid, respectively. Several compounds such as penta-*O*-galloyl-*β*-d-glucose, gallic acid, epicatechin gallate, and quercetin 3-*β*-d-glucose at high concentrations were toxic to cells, which did not induce ARE activation.

### 2.3. Cell Proliferation Assays

**Figure 3:**
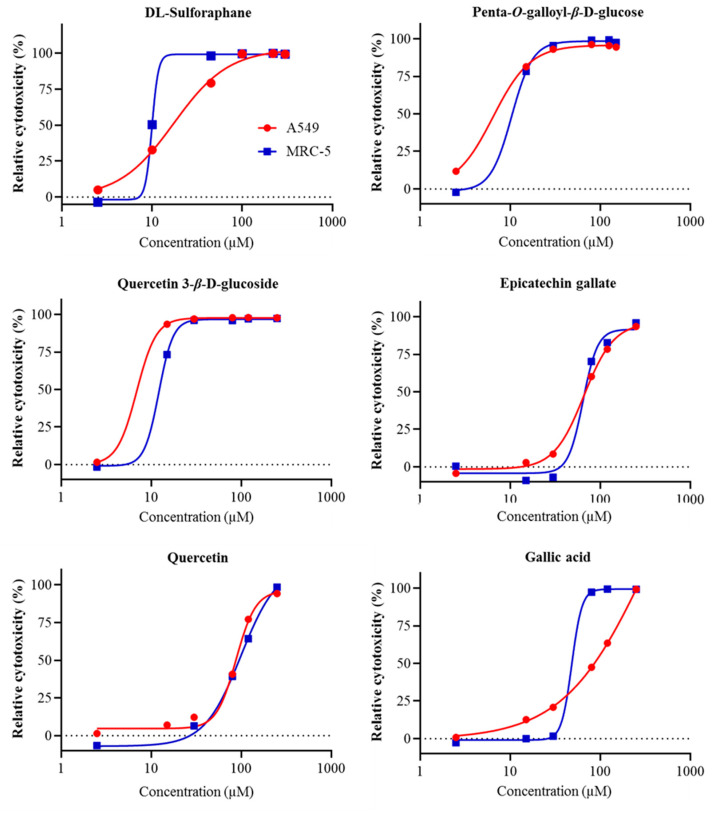
Cytotoxicity (%) of phenolic compounds (penta-*O*-galloyl-*β*-d-glucose, quercetin 3-*β*-d-glucoside, gallic acid, epicatechin gallate, ellagic acid) and the control (dl-sulforaphane) in A549 and MRC-5 cell lines. Data are expressed as percentages of cytotoxicity in A549 and MRC-5 cells treated with DMSO and the compounds compared with the corresponding vehicle controls that were A549 and MRC-5 cells treated with 0.35% DMSO only.

Cell viability assays were performed to address the cytotoxic effects of the phenolic compounds. A reduction in luminescence absorbance could result from a loss of cell viability and a reduction in cell number. The vehicle DMSO at the highest concentrations used (0.35%) did not affect cell number or viability in both A549 and MRC-5 cells, indicating that a reduction in luminescence absorbance in the presence of the tested compounds would indicate a toxic effect of these compounds rather than the vehicle. Among 16 tested compounds, penta-*O*-galloyl-*β*-d-glucose and quercetin 3-*β*-d-glucoside had the lowest IC_50_ values in the A549 cells (Table 2). The IC_50_ values of penta-*O*-galloyl-*β*-d-glucose and quercetin 3-*β*-d-glucoside in MRC-5 cells were 6.11 and 6.89 µM, respectively (Figure 3). These compounds were also toxic to the MRC-5 cells. The IC_50_ values of penta-*O*-galloyl-*β*-d-glucose and quercetin 3-*β*-d-glucoside in MRC-5 cells were 10.37 and 12.15 µM, respectively. Epicatechin gallate had IC_50_ values for A549 and MRC-5 cells that were 65.96 and 64.38, respectively, while IC_50_ values of quercetin and gallic acid for A549 and MRC-5 cells were 87.74 and 99.47, and >250 and 48.18, respectively. Other tested compounds had IC_50_ values > 250 µM in both A549 cells and MRC-5 (Table 2).

## 3. Discussion

Black walnuts have recently been documented as a promising natural source for the medicinal and pharmaceutical industries. The kernels of black walnuts have been reported to possess multiple biological functions which are likely associated with the presence of its bioactive constituents, including polyphenols. In the present study, we utilized HTS technologies to characterize the antioxidant and anticancer activities of 16 phenolic compounds found in black walnut kernels [6,9]. Given the huge availability of black walnuts, the exploration of the biological functions of the bioactive compounds in black walnuts could promote the development of novel applications of black walnut and its by-products, which could provide opportunities to utilize the abundant, low-value, renewable materials from black walnut and its by-products into profitable value-added products and thereby potentially increase the sustainability of the black walnut agro-industry.

Our results indicated that several phenolic compounds found in black walnut kernels exert strong antioxidant activities. Out of 16 phenolic compounds tested, 10 compounds (penta-*O*-galloyl-*β*-d-glucose, epicatechin gallate, quercetin, (−)-epicatechin, rutin, quercetin 3-*β*-d-glucoside, gallic acid, (+)-catechin, ferulic acid, syringic acid) exhibited higher total antioxidant capacity than Trolox. The presence of multiple phenolic compounds also raises the possibility of synergistic activities that are likely responsible for the antioxidant activities observed in black walnuts. Our results indicated that no compound can be considered to exert significant ARE induction activity since all tested compounds had ARE fold-increases relative to the control that were less than 10. Roy et al. [15] suggested that an ARE fold-increase > 10 was considered to induce the activity of ARE signaling pathways that functionally regulate the expression of genes encoding over 250 antioxidant and detoxification proteins [16]. Vu et al. [7] observed the variation of antioxidant capacities of different black walnut cultivars, in which Mystry exhibited the highest antioxidant activity among the examined cultivars. Future research will focus on purification and characterization of bioactive compounds and its composition in Mystry that mainly drive the antioxidant activity.

Our results also revealed the anticancer potential of the phenolic compounds in black walnuts. Two phenolic compounds, penta-*O*-galloyl-*β*-d-glucose and quercetin 3-*β*-d-glucoside, exhibited antiproliferative activities against both the tumorigenic alveolar epithelial cells (A549) and non-tumorigenic lung fibroblast cells (MRC-5), while no significant inhibitory effects on the growth of these cell lines were observed on other tested compounds. Black walnut and English walnut (*J. regia* L.), another common *Juglans* species, have been reported to share a similar polyphenolic profile in which all 16 phenolic compounds tested were found both in black walnut and English walnut (Table 3). The total contents of these phenolic compounds in black walnut was lower compared with English walnut [9]. Remarkably, anticancer capacities have been well established both in vivo and in vitro in English walnut. In vivo, English walnut kernels have been shown to inhibit the growth of several types of cancer cells, including colon cancer stem cells, breast cancer cells, and colorectal cancer cells [17,18,19]. Consumption of English walnut kernels by mice was associated with in vitro tumor cell changes in DNA proliferation and apoptosis [20,21,22]. Anticancer functions of English walnuts are possibly associated with its polyphenols [19,23], suggesting possible anticancer capacities of black walnuts and its polyphenols.

Vu et al. [9] reported that the contents of phenolic compounds were widely variable among different black walnut cultivars. Among the six black walnut cultivars whose antioxidant capacities have been examined, the kernel extracts from Mystry and Surprise have been documented to possess the strongest antioxidant activities [7]. Remarkably, penta-*O*-galloyl-*β*-d-glucose only presented in Mystry (15.2 mg/kg) and was not detectable in other cultivars (e.g., Surprise) showing the antioxidant activities (Table 3). The concentration of this compound in black walnut (Mystry) was lower compared with English walnut (58.6 mg/kg). Due to a huge variation in the contents of phenolic compounds in different black walnut cultivars, it is likely that the antioxidant activities of these extracts were possibly driven by not only penta-*O*-galloyl-*β*-d-glucose but also other compounds. Additionally, the presence of multiple phenolic compounds in black walnut raises the possibility of synergistic effects of these compounds that are responsible for the biological activities of black walnuts.

The results from HTS assays indicated penta-*O*-galloyl-*β*-d-glucose as a potent bioactive compound. This compound exhibited strong antioxidant and anticancer capacities. The fold-increase over Trolox of penta-*O*-galloyl-*β*-d-glucose was 11.5, whereas the IC_50_ value of this compound for the tumorigenic alveolar epithelial cell line (A549) was 6.11. Zhang et al. [10] reported 1, 2, 3, 4, 6-penta-*O*-galloyl-*β*-d-glucose, a hydrolysable tannin, as a polyphenolic compound highly enriched in several plants and herbs such as *Acer truncatum*, *Paeonia suffruticosa*, *Rhus chinensis*, *Schinus terebinthifolius*, and *Terminalia chebula*. Previous studies have documented a variety of biological functions of penta-*O*-galloyl-*β*-d-glucose, including antidiabetic, antibacterial, antioxidant, anticancer, antiangiogenic, antivirus, and anti-inflammatory activities [10,25]. This compound has been shown to possess anticancer effects against multiple cancer cells including lung cancer, prostate cancer, and breast cancer [26,27,28]. Huh et al. [28] reported antitumor activities of 1, 2, 3, 4, 6-penta-*O*-galloyl-*β*-d-glucose that primarily inhibited angiogenesis through cyclooxygenase-2 and phospho-p38 mitogen-activated protein kinase (MAPK)-dependent pathways. Additionally, Bae et al. [29] suggested 1,2,3,4,6-penta-*O*-galloyl-*β*-d-glucose as a potential candidate antiviral drug to treat varicella-zoster virus (VZV)-associated diseases (e.g., chickenpox) due to its significant suppressive inhibitory effect on VZV-induced c-Jun N-terminal kinase (JNK) activation, expression of viral immediate-early 62 (IE62) protein, and VZV replication. Vu et al. [9] quantified the amount of penta-*O*-galloyl-*β*-d-glucose in kernel extracts from 11 different black walnut cultivars and reported that the highest amount of penta-*O*-galloyl-*β*-d-glucose was found in Mystry (15.2 mg/kg). This cultivar has been documented to possess multiple bioactive activities including antioxidant, antibacterial, and anti-inflammatory potential. Future research might focus on exploring the anticancer properties of Mystry and its bioactive constituents.

## 4. Materials and Methods

### 4.1. Sample Preparation

Chemicals including (+)-catechin, (−)-epicatechin gallate, ellagic acid, ferulic acid, gallic acid, naringin, *p*-coumaric acid, *p*-hydroxybenzoic acid, penta-*O*-galloyl-*β*-d-glucose, quinic acid, quercetin, quercetin-3-*β*-d-glucoside, rutin, syringic acid, vanillic acid, Trolox, DL-sulforaphane, and tert-butylhydroquinone (TBHQ) were purchased from Sigma-Aldrich (purity ≥ 95%, Sigma-Aldrich, St. Louis, MO, USA). The compound, glansreginin A, was not included in this study since this compound was not commercially available. In all assays, all chemicals were solubilized in 100% DMSO (dimethyl sulfoxide, tissue-culture grade, Sigma-Aldrich) to facilitate acoustic transfer and were transferred acoustically to the assay plates using an Echo Liquid Handler (Echo^®^ 555, Beckman Coulter Inc., Brea, CA, USA). All compounds were evaluated for antioxidant and antitumor activities in dose-response assays at 7 concentrations of 0, 2.5, 15, 30, 80, 120, and 250 µM, except for penta-*O*-galloyl-*β*-d-glucose, Trolox, DL-sulforaphane, and TBHQ. The concentrations of penta-*O*-galloyl-*β*-d-glucose tested were 0, 2.5, 15, 30, 80, 125, and 175 µM, and Trolox was screened at concentrations of 0, 5, 10, 20, 40, 80, and 120 µM, whereas dl-sulforaphane and TBHQ were evaluated at concentrations of 0, 2.5, 10, 45, 100, 220, and 300 µM. DMSO was used for backfill and the highest final concentration of DMSO added into the cells was 0.35%. The cells treated with 0.35% DMSO and without DMSO were included as vehicle controls in the assays. In all screening assays, all compounds were tested with one replicate per concentration per assay to generate a linear curve of each activity for each compound. The vehicles and positive controls (dl-sulforaphane, TBHQ) were screened via four replicates to identify variability of data.

### 4.2. Cell Lines

An Nrf2 antioxidant response element (ARE) reporter HepG2 cell line, a stably transfected liver cell line expressing a firefly luciferase gene under the control of the ARE, was purchased from BPS Bioscience (San Diego, CA, USA). The human alveolar epithelial cell line A549 and the human lung fibroblast cell line MRC-5 were obtained from American Type Culture Collection (ATCC) (CCL-185 and CCL-171, ATCC, Manassas, VA, USA). The HepG2-ARE cells were grown in modified Eagle’s medium (MEM) supplemented with GlutaMAX, 10% fetal calf serum (FBS) and 600ug/mL Geneticin (Thermo Fisher Scientific, Waltham, MA, USA). The tumorigenic alveolar epithelial cells (A549) and non-tumorigenic lung fibroblast cells (MRC-5) were grown in RPMI medium supplemented with 10% FBS. All cells were grown and maintained at 37 °C in a humidified incubator with 5% CO_2_.

### 4.3. Total Antioxidant Capacity

The antioxidant capacity of the phenolic compounds was evaluated using a total antioxidant capacity (TAC) colorimetric assay kit (K274-100, BioVision, CA, USA), according to the manufacturer’s instructions. Briefly, the phenolic compounds tested at 7 concentrations (as described above) were added to 384-well plates. Subsequently, Cu^2+^ working solution (12.5 µL/ well) was added into the sample wells. The 384-well plates were incubated for 1.5 h at room temperature and the absorbance of the samples was then read at 570 nm using a microplate reader (Enspire, Perkin Elmer Inc., Waltham, MA, USA). Trolox was used to standardize the antioxidant capacity, as recommended by the manufacturer. A Trolox standard curve was included, and the total antioxidant capacity of the phenolic compounds was interpolated and expressed as Trolox equivalent (mM) from a seven-parameter logistic curve of the Trolox control using curve-fitting software.

### 4.4. Antioxidant Response Element (ARE) Activation

The impact of the phenolic compounds on ARE activation in the HepG2-ARE cell line was evaluated using Steady-Glo^®^ Luciferase assay system (E2510, Promega, Madison, WI, USA), following the manufacturer’s instructions. In brief, the HepG2 -ARE cells were seeded at a density of 10,000 cells/well in 384-well plates containing 50 µL of the complete media per well using a Multidrop Combi dispenser (Thermo Fisher Scientific, Waltham, MA, USA) and then the plate cultures were incubated at 37 °C in a 5% CO_2_ humidified incubator for 20 h. The HepG2-ARE cells were incubated with compounds for 18 h. The known ARE activator TBHQ was used as a positive control and the cells treated with 0.35% DMSO and without compounds tested served as a vehicle control. The cells in the absence of DMSO and the compounds were utilized for measuring the background luminescence. The reporter activity was measured by the addition of 25 µL Steady-Glo^®^ luciferase assay reagent (Promega) for 30 min using the Multidrop Combi dispenser (Thermo Fisher Scientific). The luminescence intensities of the 384-well plates were read on Enspire microplate reader (Perkin Elmer Inc.). Percent cytotoxicity of compounds was normalized to the positive and negative controls on each assay plate.

### 4.5. Cell Proliferation Assays

Influence of the phenolic compounds on cell growth in the tumorigenic alveolar epithelial cells (A549) and non-tumorigenic lung fibroblast cell (MRC-5) cell lines was investigated using the CellTiter-Glo^®^ cell viability assay kit (G7571, BioVision, CA, USA), according to the manufacturer’s instructions. Briefly, the A549 and MRC-5 cells were seeded at densities of 8000 and 3000 cells per well, respectively, in 384-well plates and were then incubated in a 5% CO_2_ humidified incubator at 37 °C. The cultures were treated with the phenolic compounds and dl-Sulphorane (a known antiproliferative agent as a positive control) at 7 final concentrations (as mentioned above) and a vehicle (0.35% DMSO). After 72h of incubation, CellTiter-Glo Luminescent assay reagent (Promega) was dispensed at a volume of 10 µL per well into the 384-well plates for 10 min using the Matrix Wellmate dispenser (Thermo Fisher Scientific). The plates were allowed to incubate at room temperature for 20 min and then the luminescence was read using an Enspire microplate reader (Perkin Elmer Inc.)

### 4.6. Data Analysis

For total antioxidant capacity analysis, linear regression analysis was performed to identify the linear regression equation for each compound using GraphPad Prism 8 (San Diego, CA, USA). The coefficient of the compound equation was compared with the coefficient of the Trolox control to determine the relative total antioxidant capacity of each compound. Fold-increase over Trolox was calculated by dividing the coefficient of the compound models by the coefficient of the Trolox control. The compounds that exhibited a fold-increase over Trolox greater than 5 were considered to possess significant total antioxidant capacity [15].

The ARE fold induction of the compounds was measured by dividing the luminescence absorbance of the treatment by the specific luminescence absorbance of the control sample and multiplying by 100. The control sample (in the presence of DMSO vehicle and without the compounds) was set at 100%. The compounds that had ARE fold induction to 10-fold over the vehicle controls in one or more concentrations were considered to have significant ARE induction activity [15].

The relative cytotoxicity (%) of the phenolic compounds was calculated by dividing the specific luminescence absorbance of the treated sampled by the specific luminescence absorbance of the control sample and multiplying by 100. The control sample (in the presence of DMSO vehicle and without the compounds) was set at 100%. Non-linear regression analysis of data was performed to identify the dose-response curve for each compound. The IC_50_ values (half maximal inhibitory concentration) of each compound were determined from the dose-response curve for the A549 and MRC-5 cell lines using GraphPad Prism 8. The compounds that exhibited IC _50_ values < 10 µM in the A549 cell line and had no toxic effects on the control cell line MRC-5 were considered to be potent antiproliferative compounds.

## 5. Conclusions

We identified the antioxidant and anticancer potentials of phenolic compounds found in black walnuts. Out of 16 tested compounds, several compounds had remarkable antioxidant activities and two compounds had strong anticancer activities. Penta-*O*-galloyl-*β*-d-glucose exhibited the strongest antioxidant and antiproliferative activities against both the tumorigenic alveolar epithelial cells (A549) and non-tumorigenic lung fibroblast cells (MRC-5) among the tested compounds. Antioxidant activities of black walnut cultivars are likely correlated with the synergistic effects of phenolic compounds in black walnut. Black walnut extracts possibly possess anticancer activity. Penta-*O*-galloyl-*β*-d-glucose has been previously documented to exert a wide range of bioactive activities. Our results support the notion that penta-*O*-galloyl-*β*-d-glucose could be a potential bioactive compound for the cosmetic and pharmaceutical industries.

## Figures and Tables

**Table 1:** Antioxidant activities of phenolic compounds in black walnut.

No.	Compound	Slope (in Trolox Equivalents)	R Square	Fold-Increase Over Trolox
**Control**				
1	Trolox	0.001014 ± 1.125 × 10^−5^	0.999	1.0
**Antioxidant Capacity Higher than Trolox**				
2	Penta-*O*-galloyl-*β*-d-glucose	0.01167 ± 5.756 × 10^−4^	0.995	11.5
3	Epicatechin gallate	0.004294 ± 1.570 × 10^−4^	0.996	4.2
4	Quercetin	0.004045 ± 1.392 × 10^−4^	0.997	4.0
5	(−)-Epicatechin	0.003729 ± 7.546 × 10^−5^	0.999	3.7
6	Rutin	0.002962 ± 1.551 × 10^−4^	0.989	2.9
7	Quercetin 3-*β*-d-glucoside	0.002908 ± 1.522 × 10^−4^	0.989	2.9
8	Gallic acid	0.002541 ± 1.392 × 10^−5^	0.999	2.5
9	(+)-Catechin	0.002029 ± 3.184 × 10^−5^	0.999	2.0
10	Ferulic acid	0.001775 ± 5.026 × 10^−5^	0.997	1.8
11	Syringic acid	0.001088 ± 1.057 × 10^−5^	0.999	1.1
**Antioxidant capacity lower than Trolox**				
12	Vanillic acid	0.0007396 ± 4.245 × 10^−5^	0.984	0.7
13	Ellagic acid	0.0005183 ± 2.860 × 10^−5^	0.988	0.5
14	Naringin	0.0005177 ± 9.755 × 10^−6^	0.998	0.5
15	*p*-Coumaric acid	0.0003139 ± 1.434 × 10^−5^	0.992	0.3
16	*p*-Hydroxybenzoic acid	−0.000008 ± 1.260 × 10^−5^	0.086	n/a
17	Quinic acid	−0.0000004 ± 1.034 × 10^−6^	0.032	<0.1

**Table 2:** Half maximal inhibitory concentrations (IC_50_) of phenolic compounds (µM) in black walnuts in A549 and MRC-5 cell lines.

Compound	A549 Cell Line	MRC-5 Cell Line
Penta-*O*-galloyl-*β*-d-glucose	6.11	10.37
Quercetin 3-*β*-d-glucoside	6.89	12.15
Epicatechin gallate	65.96	64.38
Quercetin	87.74	99.47
Gallic acid	>250	48.18
Ellagic acid	>250	>250
(−)-Epicatechin	>250	>250
Rutin	>250	>250
(+)-Catechin	>250	>250
Ferulic acid	>250	>250
Syringic acid	>250	>250
Vanillic acid	>250	>250
Naringin	>250	>250
*p*-Coumaric acid	>250	>250
*p*-Hydroxybenzoic acid	>250	>250
Quinic acid	>250	>250
dl-Sulforaphane (control)	16.96	9.95

**Table 3:** Concentrations of phenolic compounds (µg/g of dry weight) in kernels of six black walnut cultivars with their known bioactive activities [5,6,7,9,24].

Compound	Black Walnut Cultivar						English Walnut ^+^
	Daniel	Hay	Jackson	Kwik Krop	Mystry	Surprise	
Penta-*O*-galloyl-*β*-d-glucose	n/d	n/d	n/d	n/d	15.2 ± 2.5	n/d	55.9 ± 7.7
Quercetin 3-*β*-d-glucoside	n/d	3.2 ± 0.1	1.6 ± 0.2	n/d	2.1 ± 0.3	1.8 ± 0.3	3.7 ± 0.2
Epicatechin gallate	13.2 ± 0.5	7.0 ± 0.6	3.6 ± 0.7	n/d	2.0 ± 0.2	6.0 ± 0.7	4.9 ± 1.5
Gallic acid	n/d	1.4 ± 0.2	0.7 ± 0.2	0.5 ± 0.03	4.3 ± 0.3	1.0 ± 0.03	8.1 ± 0.7
Ellagic acid	30.4 ± 1.0	40.5 ± 5.9	61.1 ± 3.7	11.4 ± 2.0	65.7 ± 4.8	72.1 ± 8.3	98.4 ± 20.6
Rutin	n/d	n/d	n/d	1.7 ± 0.6	4.2 ± 1.3	n/d	2.7 ± 0.3
(+)-Catechin	n/d	n/d	n/d	n/d	n/d	0.6 ± 0.01	47.9 ± 3.5
Ferulic acid	n/d	n/d	n/d	0.7 ± 0.04	0.6 ± 0.06	4.9 ± 0.08	0.9 ± 0.1
Syringic acid	7.3 ± 1.3	n/d	7.7 ± 1.4	n/d	9.5 ± 2.1	n/d	7.3 ± 2.2
Vanillic acid	n/d	9.9 ± 2.6	8.7 ± 1.7	n/d	6.9 ± 1.2	n/d	7.3 ± 1.8
Naringin	0.5 ± 0.1	n/d	n/d	0.3 ± 0.1	0.5 ± 0.2	4.9 ± 0.1	0.3 ± 0.04
*p*-Coumaric acid	n/d	0.2 ± 0.03	0.2 ± 0.03	0.2 ± 0.1	0.3 ± 0.02	0.3 ± 0.1	0.5 ± 0.1
*p*-Hydroxybenzoic acid	n/d	n/d	n/d	n/d	n/d	n/d	1.2 ± 0.5
Quinic acid	4.7 ± 0.2	2.4 ± 0.1	1.4 ± 0.2	4.2 ± 0.2	2.4 ± 0.3	3.9 ± 0.5	6.8 ± 0.4
Quercetin	n/q	n/q	n/q	n/q	n/q	n/q	n/q
(−)-Epicatechin	n/q	n/q	n/q	n/q	n/q	n/q	3.9 ± 0.01
**Biological Activity of Kernel Extracts from Black Walnuts**							
Antioxidant	++	+	++	++	+++	+++	n/c
Antibacterial *	.	.	.	.	+++	+++	n/c
Anti-inflammatory potential **	+	n/a	n/a	n/a	+	+++	n/c

+: phenolic contents in an English walnut cultivar [9,24]; * antibacterial activities of the extracts against a Gram-positive bacterium (*Staphylococcus aureus*) [6]; ** : overall cytokine suppressive activities of the extracts on six cytokines/chemokines in (TNF-α, IL-1β, IL-6, IL-8, IL-10, and MCP-1) in human promonocytic cell line U-937 [5]; n/d: not detected; n/q: identified but not quantified; n/a: not reported; n/c: not comparable since the biological activities of English walnut and black walnut were not reported from the same studies; +: possessing activity; .: no activity.

## Supplementary Materials

The following are available online. Figure S1: Data distribution of controls (Trolox, DL-sulforaphane, tert- butylhydroquinone) in total antioxidant capacity and antioxidant response element (ARE) activation assays. Figure S2: Data distribution of controls (Trolox, DL-sulforaphane) in cytotoxicity assays.
