# Supplementary material for: Profiling Anticancer and Antioxidant Activities of Phenolic Compounds Present in Black Walnuts (Juglans nigra) Using a High-Throughput Screening Approach
Source: Molecules. 2020 Oct 2;25(19):4516. doi: 10.3390/molecules25194516 (PMC7583942; doi:10.3390/molecules25194516)
Supplement: Supplementary file 1 [file molecules-25-04516-s001.pdf]

Supplementary materials

Profiling Anticancer and Antioxidant Activities of Phenolic Compounds in Black Walnuts (*Juglans nigra*) using a High-Throughput Screening Approach

Khanh-Van Ho[1,2], Anuradha Roy[3], Sarah Foote[4], Phuc H. Vo[1], Namrita Lall[1,5] and Chung-Ho Lin[1*]

[1] Center for Agroforestry, School of Natural Resources, University of Missouri, Columbia, Missouri, United States

[2] Department of Food Technology, Can Tho University, Can Tho, Vietnam

[3] High Throughput Screening Laboratory, University of Kansas, Lawrence, Kansas, United States

[4] CEVA Biomune, Lenexa, Kansas, United States

[5] Department of Plants and Soil Sciences, Plant Science Complex, University of Pretoria, Pretoria, South Africa

* Correspondence: Chung-Ho Lin: LinChu@missouri.edu

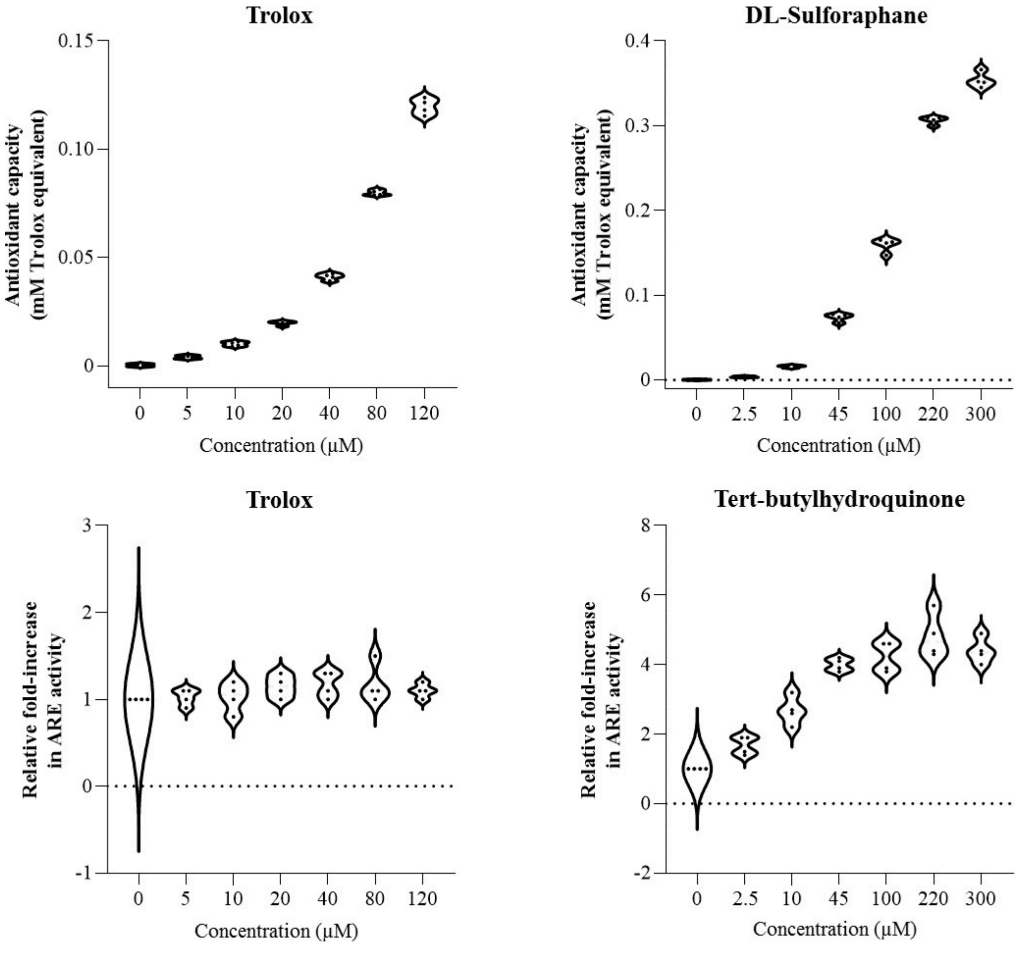

Supplementary Figure 1. Data distribution (n=4) of controls (Trolox, DL-sulforaphane, tert-butylhydroquinone) in total antioxidant capacity and antioxidant response element (ARE) activation assays. Each violin plot represents the distribution of data for each treatment/concentration. Dot symbols inside violin plots represents data points of each replicate.

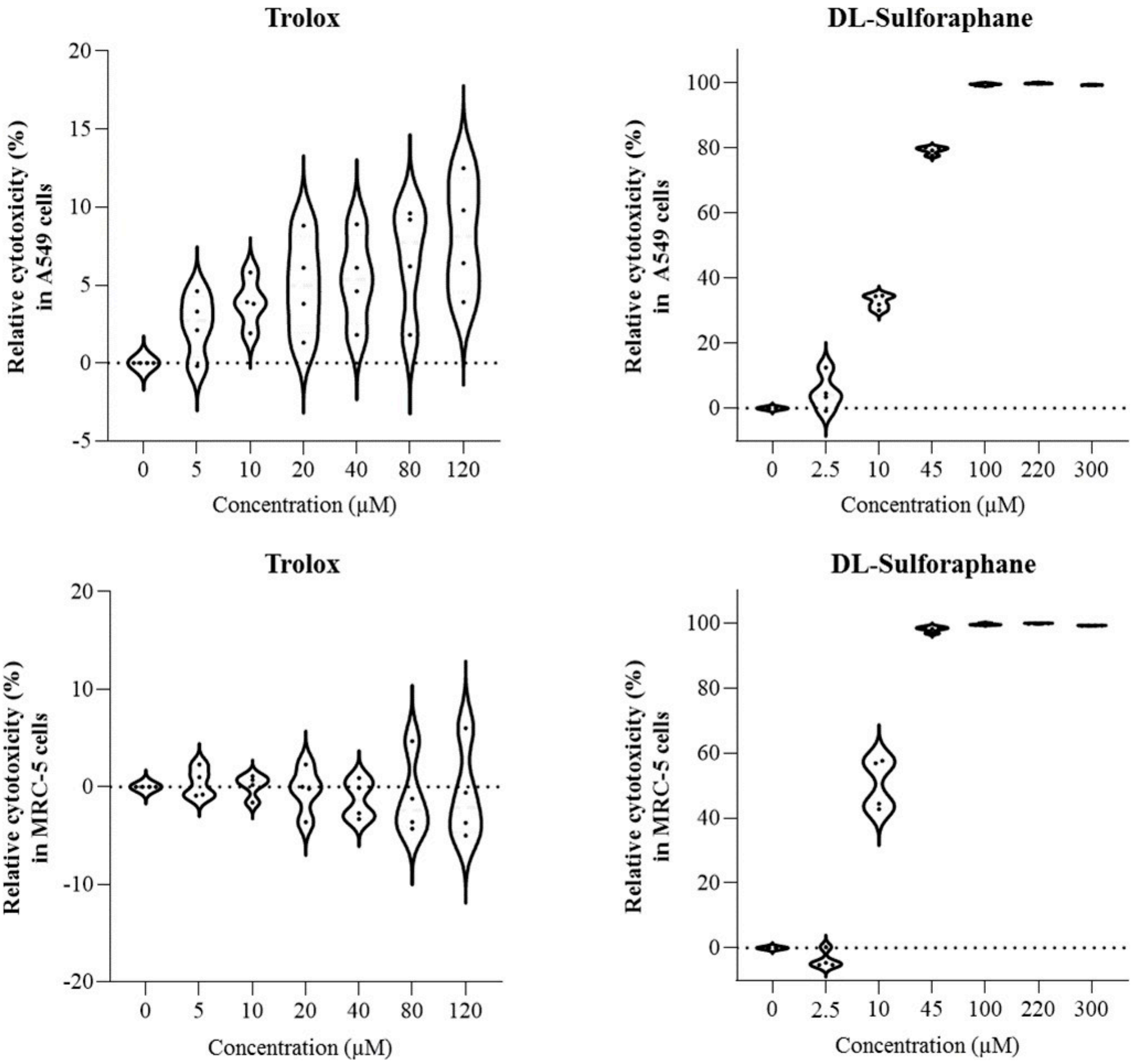

Supplementary Figure 2. Data distribution (n=4) of controls (Trolox, DL-sulforaphane) in cytotoxicity assays. Each violin plot represents the distribution of data for each treatment/concentration. Dot symbols inside violin plots represents data points of each replicate.
